# Daring to Do

**Published:** 1877-10

**Authors:** 


### DARING TO DO.

SMALL minds spend a good deal of time in deciding, as to a particular course of conduct; whether "they can afford to do it." "What will Mrs. Grundy say?" is a question of momentous interest. To do anything which Mrs. Upstart or "the Smiths" would consider "mean," is no more to be thought of, than committing a petty larceny, and being found out. It is known that any of the Wanttobe's would almost as lief be found coming out of a hen-roost at midnight, as to live in any street having "East" attached to it; while there are those who feel forty feet higher, by reason of their being able to say, "I live in Fifth Avenue;" and for such to be seen with a bundle or package in the hand on Broadway! they would fairly tremble in their shoes, lest they might be recognized by some one into whose "set" they were aiming to obtain an *entrée*.

A Baltimore Buonaparte surprised a friend one day, by carrying a broom homeward. "Why, it belongs to me!" was the reply to a question and look of incredulity. Says a Washington letter writer, "Yesterday, I saw a U. S. Senator carrying, like Lord Napier, his own small bundle, with its clean shirt and towel, its piece of soap and hair-brush." Let the young and all remember, that it is the motive which constitutes the meanness, or otherwise, of an act which is not in itself dishonorable. Better is it for a man to do a thing for himself, than to have another do it for him, when he cannot afford to pay for the service. The first step towards implanting in the mind of a child, a feeling of self-reliance and a manly independence, is to teach that child to help himself whenever it is practicable.

